# Changes in Cardiac Function During the Development of Uremic Cardiomyopathy and the Effect of Salvianolic Acid B Administration in a Rat Model

**DOI:** 10.3389/fvets.2022.905759

**Published:** 2022-06-16

**Authors:** Danfu Ma, Ahmed S. Mandour, Ahmed Elfadadny, Hanan Hendawy, Tomohiko Yoshida, Hussein M. El-Husseiny, Koji Nishifuji, Ken Takahashi, Zhenlei Zhou, Yanbing Zhao, Ryou Tanaka

**Affiliations:** ^1^College of Veterinary Medicine, Nanjing Agricultural University, Nanjing, China; ^2^Departments of Veterinary Surgery, Faculty of Veterinary Medicine, Tokyo University of Agriculture and Technology, Tokyo, Japan; ^3^Department of Animal Medicine (Internal Medicine), Faculty of Veterinary Medicine, Suez Canal University, Ismailia, Egypt; ^4^Laboratory of Veterinary Internal Medicine, Division of Animal Life Science, Institute of Agriculture, Graduate School, Tokyo University of Agriculture and Technology, Tokyo, Japan; ^5^Department of Animal Internal Medicine, Faculty of Veterinary Medicine, Damanhour University, Damanhour, Egypt; ^6^Department of Veterinary Surgery, Anesthesiology, and Radiology, Faculty of Veterinary Medicine, Suez Canal University, Ismailia, Egypt; ^7^Department of Surgery, Anesthesiology, and Radiology, Faculty of Veterinary Medicine, Benha University, Benha, Egypt; ^8^Department of Pediatrics and Adolescent Medicine, Juntendo University Graduate School of Medicine, Bunkyo-Ku, Tokyo, Japan

**Keywords:** intraventricular pressure gradients, uremic cardiomyopathy, hypertrophy, strain, salvianolic acid

## Abstract

**Background:**

Uremic cardiomyopathy (UC), the main cause of death in progressive chronic kidney disease (CKD), is characterized by diastolic dysfunction. Intraventricular pressure gradients (IVPG) derived from color m-mode echocardiography (CMME) and two-dimensional speckle tracking echocardiography (2DSTE) were established as novel echocardiographic approaches for non-invasive and repeatable assessment of cardiac function. Previously, salvianolic acid B (Sal B) showed the potential to alleviate concentric LV hypertrophy in the pressure overload model. The purpose of this study was to evaluate the changes in cardiac function in UC and assess the efficacy of Sal B therapy using IVPG and 2DSTE techniques.

**Materials and Methods:**

Twenty-four rats underwent subtotal nephrectomy to produce progressive renal failure and were allocated equally into UC (*n* = 12) and Sal B-UC (*n* = 12) groups and monitored for 8 weeks. A sham-operated group was also included in this study (*n* = 12). Sal B was injected from weeks 4 to 8 in the Sal B-UC group. Conventional echocardiography, 2DSTE, and CMME were performed every 2 weeks post-operation, concomitantly with an evaluation of renal function. Histopathological and immunohistochemistry analyses were carried out to confirm the echocardiography findings.

**Results:**

Renal failure and myocardial dysfunction were confirmed in the UC group from weeks 2 through 8. Eccentric and concentric hypertrophy was observed in the UC group, while the Sal B-UC group showed only eccentric hypertrophy. IVPG analysis did not reveal any significant differences between the groups. Edema, inflammation, fibrosis, and immunohistochemical expression of CD3 infiltration were higher in the UC group compared with sham and Sal B-UC groups.

**Conclusion:**

2DSTE and IVPG explored the pathophysiology during the development of UC and indicated the incidence of myocardial dysfunction before ventricular morphological changes without intracardiac flow changes. This study confirmed increased ventricular stiffness and fibrosis in UC rats which was potentially treated by Sal B *via* decreasing edema, inflammation, and fibrosis.

## Introduction

Cardiovascular diseases are the leading cause of death in renal failure patients (1), with hypertrophy of the left ventricle (LV) the most prevalent cardiac disorder in this population. Left ventricular hypertrophy is closely related to heart failure (2). Although scientists termed cardiomyopathy related to renal failure as uremic cardiomyopathy (UC) in 1967, the pathogenesis of UC remains poorly understood due to its multifactorial etiology (3–5).

Multiple pathological pathways are involved in the development of UC. The UC-associated LV hypertrophy results from complex pressure overload, volume overload, and the uremic state itself. Systemic hypertension results in LV pressure overload, while LV volume overload occurs due to hypervolemia and anemia (6). While Pressure overload leads to concentric LV hypertrophy (7), volume overload results in eccentric LV hypertrophy. Hypertrophy of the LV is a beneficial adaptive response during the early stages of UC, but progressive LV overload leads to maladaptive cardiomyocyte alterations and death (4). The loss of cardiomyocytes leads to LV dilatation and eventually systolic dysfunction (3). Thus, different types of LV hypertrophy are observed in UC based on the forces acting on the LV (8).

Although conventional echocardiography enables longitudinal assessments of cardiac function, it lacks the sensitivity required for detecting diastolic dysfunction (9). Intraventricular pressure gradients (IVPG), the pressure gradients inside the ventricle that draw blood from the left atrium (LA) to the LV during diastole, have been strongly correlated with left atrial (LA) pressure and active relaxation (10). Our previous research proved that IVPG could be used as a serial cardiac function evaluation tool in rodent and dog models (11–13).

Strain is a dimensionless characteristic for measuring relative deformation. Two-dimensional speckle tracking echocardiography (2DSTE) enables the quantification of both regional strain and strain rate, resulting in promising novel parameters for describing myocardial function (14).

Currently, angiotensin-converting-enzyme inhibitors and Angiotensin II Receptor Blockers are used as the first-line therapeutic drugs in UC to counteract the RAAS effect. But the drawbacks of these drugs like hypotension, azotemia, and fatigue caught the attention (15). Nowadays, there is growing interest in herbal medicine as a replacement or supportive cardioprotective treatment. Salvianolic acid B (Sal B), a water-soluble active component of *Salvia miltiorrhiza Bunge*, also known as Danshen, is widely used in Asia to treat cardiovascular disease. Sal B is well tolerant in the general population (16) and could alleviate cardiac fibrosis *in vitro* and improve myocardial function in diabetic cardiomyopathy (17, 18). Sal B was proved to decrease the inflammation and fibrosis induced by the Angiotensin II (17), so anti-inflammation and antifibrosis are considered the main treatment mechanism in cardiovascular diseases (19). Recently, we demonstrated that 5 mg/kg Sal B comparatively improves cardiac function in rats with experimental LV hypertrophy (12). We select 5 mg/kg as the proper dose to treat UC based on a previous study because this dose showed proper effect and duration (20). To the best of our knowledge, no studies have investigated the utility of combining IVPG and 2DSTE in UC. In addition, the effect of Sal B on UC hemodynamics, myocardial function, and morphology is still unknown. In the current experiment, we investigated the usefulness of IVPG and 2DSTE in a UC model and explored the effect of Sal B as a potential treatment for UC. We hypothesized that different types of LV hypertrophy might contribute to the development of UC and that Sal B may alleviate the adverse effects of UC in the rat model.

## Materials and Methods

### Animals and Ethical Approval

The experiment was conducted with 36 female Sprague Dawley rats, aged 3 months and weighing between 210 g and 250 g. All procedures followed the Guide for the Care and Use of Laboratory Animals (1994) and were approved by the Institutional Animal Care and Use Committee of the Tokyo University of Agriculture and Technology (Approval No. 31-36). The rats had free access to food and water and were housed at 20°C with a 12 h light/dark cycle.

### Induction of Uremic Cardiomyopathy

A 5/6 nephrectomy was performed according to previously described procedures (21). Briefly, the rats were transferred into an anesthetic induction chamber, anesthesia was induced with 5% isoflurane until the rats reach deep anesthesia, then pentobarbital (40 mg/kg) was intraperitoneal injected. The left kidney was exposed by a left laparotomy under a surgical microscope (Leica M60, Wetzlar, Germany). The upper and lower kidney poles were electronically cauterized by a monopolar electrosurgical generator (Volleylab Force FX electro surgical Unit, Dublin, Ireland) and two 1 mm^2^ Spongy Gelatin Absorbent (Huachen, Jiangxi, China) on the kidney bipolar wound, then the abdominal wall was closed. Because the whole surgery would take a proficient operator 25 min to complete and the effect of pentobarbital last more than 1 h, we provided two bags for the operated rats to prevent intra- and post-operative hypothermia until the anesthesia wore off. For intra- and post-operative pain management, butorphanol (0.5 mg/kg) and midazolam (0.5 mg/kg) was administrated if we observed pain after the surgical procedures (22). Signs of pain include hunched over ruffled, disheveled fur, squinty eyes, dull corneas, nose to the floor, and no eating or drinking. One week later, a total right nephrectomy was performed. The nephrectomized rats were divided into two groups: UC rats (*n* = 12) and Sal B-UC treated rats (*n* = 12); 12 rats were sham-operated and served as a control group.

### Treatments

The Sal B-UC group was treated with Sal B from weeks 4 to 8 post-operation. Daily, 5 mg/kg of Sal B (Danshen DuofensuanYan 100 mg, GreenValley Inc, Shanghai, China) was injected into the abdominal cavity after the rats were tightly caged (Supplementary Figure S1). The UC rats received the same volume of normal saline by injection to limit the disturbance caused by the injection on the experimental results.

### Blood Sampling and Renal Function Tests

Blood samples were drawn from every rat at weeks 2, 4, 6, and 8. After the rats were tightly caged, 2 ml of blood was collected from the tail vein using plain tubes. Blood samples were centrifuged at 3,500 rpm for 5 min, and clean non-hemolyzed serum was kept for analysis. The concentration of serum creatinine and blood urea nitrogen (BUN) were measured automatically by special kits (v-CRE-P and v-BUN-P, Fujifilm, Tokyo, Japan) and a Chemistry Analyzer (Dri-chem 7000V, Fujifilm, Tokyo, Japan).

### Blood Pressure

Blood pressure was monitored using the oscillometric method (BP monitor for rats, Muromachi, Japan). The cuff was placed over the base of the rat's tail when the rat was tightly caged. Measurements were performed three times, and the average systolic, diastolic, and mean arterial blood pressures were recorded.

### Echocardiography

Echocardiography was performed 2, 4, 6, and 8 weeks after right nephrectomy (ProSound F75 premier CV, Hitachi Healthcare System Inc, Tokyo, Japan). Isoflurane (2.5%) was administrated to rats by mask. Then we put the rats' right recumbency for short-axis echocardiograph parameter measurements, including IVSd, LVIDd, LVPWd, IVSs, LVIDs, and LVPWs, using a 1–15 Mhz transducer (UST-52129, Hitachi Healthcare System Inc, Tokyo, Japan). The left ventricle mass (LVM) and relative wall thickness (RWT) were calculated with the following formulas (12):

LVM = [(LVIDd + LVPWd + IVSd)^3^- LVIDd^3^] ^*^1.04

RWT = (IVSd + LVPWd)/LVIDd

### Tissue Doppler Imaging and CMME for IVPG Analysis

Tissue Doppler imaging of the left parasternal (apical view) was performed, sampling transmitral E A flow velocity, E′ septum(mitral valve root velocity), and E′ lateral. The following formula was used to calculate E/E′:

E/E′ = (E/E′ lateral + E/E′ septum)/2

Color M-mode echocardiography (CMME) was performed in the transmitral flow window. To capture mitral inflow, the sample volume spanned from the orifice of the mitral valve to the LV apex, and the time-motion relation was sampled after the baseline changed to −64. The CMME image was analyzed using MATLAB (The MathWorks, Natick, MA). Prior machine settings (12) and an analysis algorithm have been described previously (23). Total IVPG was divided into two sections; Firstly, from the base of the LV near the mitral valve to one-third of the LV was termed the basal IVPG, and secondly, the gradient from the apex to two-thirds of the LV was termed the mid-to-apical IVPG (Supplementary Figure S2).

### Speckle Tracking Echocardiography

Loops of LV movement in four chambers (apical views) were acquired. Speckle tracking analysis was performed using an algorithm incorporated into EchoPAC PC DAS-RSI (Hitachi Aloka Co., Tokyo, Japan). The endocardium was traced manually for both end-systole and end-diastole phases. The software algorithm automatically divided each imaging plane of the LV into three equally circular sections: basal, midventricular, and apex on the septal and lateral aspects (Supplementary Figure S3). The longitudinal strain rate was obtained in six sections (14).

### Histological Analysis

Heart tissue samples were collected from the three groups at the end of the study (weeks 8). We sectioned (4-μ*m*), fixed the heart tissue samples in 10% neutral buffered formalin, and then embedded them in paraffin wax. The sections were cut, deparaffinized, rehydrated and then stained with Masson's trichrome and hematoxylin, and eosin (H&E). For statistical measurements, 36 sections (12 sections per group, each 4-μ*m* thick) were used. The histopathological images were examined using image software (CellSens Standard; Olympus, Tokyo, Japan). The quantification of the histopathological score has been reported in detail before (24). Briefly, 36 sections (12 sections per group) were analyzed blindly by two histopathologists based on inflammatory cells infiltration (mononuclear cell), edema (interstitial edema between the cardiocytes), necrosis (degeneration and necrotic area), and myocytes arrangement (assembled or disassembled and direction of myocardial cells). The results of both investigators were averaged for all sections in the three groups and graded as 0 (none), 1 (mild), 2 (moderate), 3 (severe), or 4 (very severe) based on mononuclear cell infiltration, edema, necrosis, and myocyte arrangement. Fibrosis intensity was assessed at the interstitial level and perivascular area.

### Immunohistochemical Staining of CD3

The same number of sections from each group were deparaffinized and rehydrated. Antigen retrieval was performed by incubating slides for 15 min at 95 °C in Tris-EDTA buffer (*pH* 9.0). Peroxidase blocking was achieved by treatment with 0.3% H_2_O_2_ diluted in methanol for 15 min. Nonspecific protein binding was inhibited by incubating the samples with 5% goat serum diluted in phosphate buffer saline with 0.1% Tween 20 for 2 h at room temperature, followed by overnight incubation with primary antibodies against monoclonal CD3 antibody (1:100, Invitrogen PA5-88511) at 4 °C. On the second day, the sections were incubated with horseradish peroxidase-conjugated secondary antibody for 1 h at room temperature. Antigen-antibody complexes were visualized after staining the samples with 3, 3′-diaminobenzidine. Cell nuclei were counterstained with hematoxylin. The frequency of CD3 cell infiltration was quantified for each of the three groups as the average number of CD3+ cells per 1 mm^2^ of tissue.

### Statistical Analysis

Data analysis was performed with a two-way analysis of variance (ANOVA) test with *post-hoc* contrasts by Tukey's honestly significant difference test using SPSS 26.0 (SPSS, Inc., Chicago, IL). The data are presented as mean ± standard deviation for all obtained parameters, and *p* < 0.05 were considered statistically significant.

## Results

### Clinical Symptoms

Polyuria was defined as bedding being more wet than usual at routine cleaning. Polyuria was observed in the UC group 4 weeks after the operation. Polyuria was more evidenced in the UC group at 6 and 8 weeks (11/12, 12/12, respectively) than Sal-B (7/12, 6/12, respectively). Besides, swelling in the abdomen and limbs was noticed from 6 weeks in 50 % and 75% of rats from the UC group at 6 and 8 weeks, respectively, which indicated hypervolemia in the UC group. In contrast, no rats from sham and Sal-B groups showed clinical signs of swelling.

### Renal Function Test

**Figure 1:**
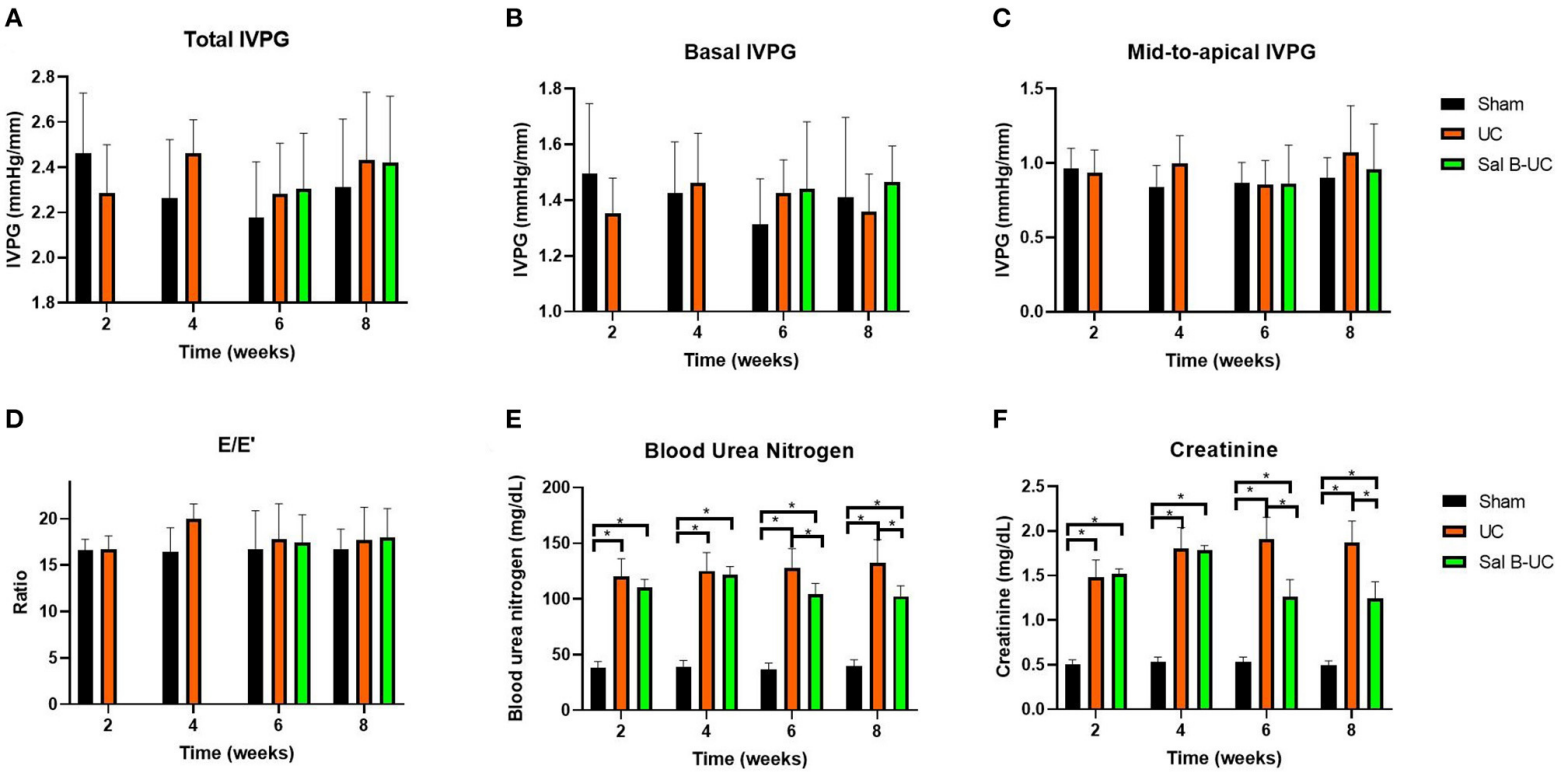
Novel echocardiography and kidney function in uremic cardiomyopathy, sham operation, and Sal B-treated rats. Novel echocardiographic measurements in the sham, UC, and Sal B-UC groups. Two-way ANOVA was performed to test the differences between groups and time points, Tukey's *post-hoc* test used group comparisons. * Indicate a significant difference between groups, *p* < 0.05. **(A)** Total IVPG, the mix of basal and mid-to-apical intraventricular pressure gradients. **(B)** Basal IVPG, the basal intraventricular pressure gradients. **(C)** Mid-to-apical IVPG, the mid plus apex intraventricular pressure gradients. **(D)** E/E′, ratio of E to E′. **(E)** creatinine. **(F)** BUN, blood urea nitrogen.

A significant increase (*p* < 0.001) in serum creatinine and BUN was observed in UC rats at the two- and four-week time points (Figure 1). Also, these biomarkers were significantly higher (*p* < 0.001) in the UC group than in the Sal B-UC group; nevertheless, serum creatinine and BUN were higher in the Sal B-UC group than in the sham group. And the percentage change analysis was shown in the Supplementary Data.

### Conventional Echocardiography

Table 1 illustrates the cardiac morphology of the sham, UC group at 6 and 8 weeks in Table 1. Data collected at 2 and 4 weeks from sham and UC groups are presented in Supplementary Tables S1A. The UC group had higher IVSd, LVPWd, IVSs, LVPWS, and FS, but lower LVIDd and LVIDs, compared with the sham group. Moreover, the LVM and RWT were increased in the UC rats at all experimental intervals compared with the sham group. Based on these results, we confirmed that significant concentric LV hypertrophy occurred in the early stages of UC development. No difference was detected between the UC and Sal B-UC groups in IVSd, LVPWd, IVSs, LVPWS, FS, and LVIDs at the 6- and 8-week time points. Also, the observed IVSd and LVPWd values in the Sal B-UC group were < in the UC group but were still > in the sham group. In contrast, LVIDd, LVPWs, and FS were higher in the Sal B-UC group than in the UC group. Concentric hypertrophy was observed in the UC group at the two-, 6-, and 8-week time points because the LVM and RWT were significantly (*p* = 0.001 and *p* < 0.001) higher compared to the sham group. We noticed eccentric hypertrophy in the UC group at 4 weeks and in the Sal B-UC group at weeks 6 and 8. The LVM was increased in all groups at 8 weeks but the UC-SalB group showed the lowest value. These results indicate that Sal B delays the progressive development of hypertrophy.

**Table 1:** Cardiac morphology of the investigated groups at 6 and 8 weeks.

**Time**	**6 weeks**			**8 weeks**			***p* of group**	***p* of time**
**Group**	**Sham**	**UC**	**Sal B-UC**	**Sham**	**UC**	**Sal B-UC**		
IVSd, mm	1.21 ± 0.17	1.57 ± 0.35^*^	1.39 ± 0.26	1.31 ± 0.16	1.67 ± 0.3^*^	1.57 ± 0.15^†^	0.000^**^	0.073
LVIDd, mm	7.53 ± 0.62	7.32 ± 0.27	7.93 ± 0.5^‡^	7.25 ± 0.58	7.32 ± 0.54	7.87 ± 0.69^‡^	0.007^**^	0.423^*^
LVPWd, mm	1.32 ± 0.27	1.78 ± 0.54^*^	1.43 ± 0.36	1.64 ± 0.33	1.95 ± 0.53	1.49 ± 0.46	0.004^**^	0.087
IVSs, mm	2.06 ± 0.39	2.29 ± 0.55	2.16 ± 0.46	1.96 ± 0.4	2.5 ± 0.52^*^	2.63 ± 0.19^†^	0.000^**^	0.042^*^
LVIDs, mm	4.34 ± 0.5	4.25 ± 0.38	4.48 ± 0.36	4.36 ± 0.46	3.93 ± 0.51^†^	4.13 ± 0.44	0.029^*^	0.028^*^
LVPWs, mm	2.32 ± 0.32	2.55 ± 0.72	2.39 ± 0.18	2.38 ± 0.43	2.88 ± 0.68^*^	2.95 ± 0.44^†^	0.009^**^	0.001^**^
FS %	42.59 ± 4.63	41.78 ± 4.99	43.58 ± 4.19	39.59 ± 7.6	46.45 ± 5.41^*^	47.67 ± 3.28^†^	0.003^**^	0.157
LVM (gm)	0.62 ± 0.11	0.91 ± 0.34^*^	0.79 ± 0.23	0.71 ± 0.11	0.99 ± 0.38	0.86 ± 0.24	0.011^*^	0.151
HW(g)	0.85 ± 0.09	1.17 ± 0.12^*^	1.09 ± 0.12^†^	0.88 ± 0.12	1.37 ± 0.12^*^	1.19 ± 0.12^‡^^†^	0.000^**^	0.001^**^
HW/BW(mg/g)	3.62 ± 0.19	5.11 ± 0.29^*^	4.61 ± 0.27^‡^^†^	3.62 ± 0.24	5.61 ± 0.31^*^	4.51 ± 0.34^‡^^†^	0.000^**^	0.000^**^
RWT	0.34 ± 0.07	0.46 ± 0.13^*^	0.36 ± 0.07^†^^‡^	0.41 ± 0.07	0.49 ± 0.09^*^	0.39 ± 0.06	0.000^**^	0.045^*^

*Echocardiographic measurements and longitudinal strain rate in the sham, UC, and Sal B-UC groups. Two-way ANOVA was performed to test the difference between groups and time points, and Tukey's post-hoc tests were used for group comparisons. Significance marks were fitted to compare data at each time point*.

* *Indicates a significant difference between the sham and UC groups. The*

** *symbol indicates the significant difference of p < 0.01*.

† *Indicates a significant difference between Sham and Sal B-UC groups*.

‡ *Indicates a significant difference between the UC and UC-Sal B groups. The significance level was p < 0.05. IVSd, interventricular septum diastolic diameter; LVIDd, left ventricular internal diastolic diameter; LVPWd, left ventricular posterior wall diastolic diameter; FS, fraction shorting; LVM, left ventricle mass; RWT, relative wall thickness; IVSs, interventricular septum systolic diameter; LVIDs, left ventricular internal systolic diameter; LVPWs, left ventricular posterior wall systolic diameter; FS, fraction shorting; LVM, left ventricle mass; RWT, relative wall thickness; HW, heart weight (g); HW/BW, heart weight (mg)/body weight (g). In this table, the two-way anova only include the data in 6 and 8 weeks. The full data including 2 and 4 weeks could be seen at Supplementary Table S3*.

### Blood Pressure and Doppler Hemodynamic Results

Mitral inflow and TDI data are summarized in Table 2. Hemodynamic data collected at 2 and 4 weeks are presented in the Supplementary Table S1B. The HR in the UC group was higher than in the sham group at every time point. UC group showed higher blood pressure than the sham group. The Sal B-UC group showed lower systolic, diastolic, and mean arterial blood pressure than the UC. Systolic arterial blood pressure in the UC rats was higher than in the sham rats from the second week (*p* < 0.001). Also, no significant differences were detected in E, E', and E/E' between the three groups. IVPG data is illustrated in Figure 1. No difference was detected between the sham, UC, and Sal B-UC groups in total, basal, and mid-to-apical IVPG.

**Table 2:** Blood pressure and Doppler hemodynamic measurements in rats at 6 and 8 weeks.

**Time**	**6 weeks**			**8 weeks**			***p* of group**	***p* of time**
**Group**	**Sham**	**UC**	**Sal B-UC**	**Sham**	**UC**	**Sal B-UC**		
HR, BPM	298.74 ± 47.14	378 ± 56.29^*^	353.03 ± 42.92‡	310.23 ± 50.05	375.72 ± 45.1^*^	364.51 ± 45.82^‡^	0.000^**^	0.605
SAP, mmHg	93.41 ± 15	133.58 ± 13.56^*^	121.24 ± 9.45^†^	100.53 ± 15.25	144.12 ± 11.98^*^	126.75 ± 11.21^†^^‡^	0.000^**^	0.134
DAP, mmHg	73.75 ± 6.55	104.13 ± 9.8^*^	88.59 ± 9.82^†^^‡^	73.2 ± 5.63	123.93 ± 19.57^*^	106.18 ± 14.43^†^^‡^	0.000^**^	0.000^**^
MAP, mmHg	80.3 ± 6.17	113.94 ± 8.08^*^	110.36 ± 7.15^†^	82.31 ± 8.05	130.66 ± 13.52^*^	119.89 ± 8.4^†^^‡^	0.000^**^	0.000^**^
E, cm/s	105.58 ± 17.51	106.49 ± 10.79	103.26 ± 14.52	97.71 ± 16.91	106.47 ± 16.2	99.83 ± 19.44	0.054	0.371
E', cm/s	5.18 ± 1.06	6.32 ± 1.42^*^	6.06 ± 1.09	5.48 ± 0.6	6.16 ± 0.77	5.62 ± 0.53	0.024	0.674

*The echocardiographic measurements of the sham and UC groups. Two-way ANOVA was performed to test the difference between groups and time points, and Tukey's post-hoc tests were used for group comparisons. Significance marks were fitted to compare data at each time point*.

* *Indicates a significant difference between the sham and UC groups, The*

** *symbol indicates the significant difference of p < 0.01*,

† *Indicates a significant difference between Sham and Sal B-UC groups*,

‡ *Indicates a significant difference between the UC and Sal B-UC groups. The significance level was p < 0.05. HR, heart rate; SAP, systolic arterial pressure; DAP, diastolic arterial pressure; MAP, mean arterial pressure; E, the velocity of early mitral inflow; E′, Peak velocity of early diastolic mitral annular motion as determined by pulsed-wave Doppler. In this table, the two-way anova only include the data in 6 and 8 weeks. The full data including 2 and 4 weeks could be seen at Supplementary table S4*.

### Speckle Tracking Echocardiography

The strain rates based on speckle tracking echocardiography are shown in Table 3. Strain rates collected at 2 and 4 weeks from sham and UC groups are presented in the Supplementary Table S1C. Compared with the sham group, the UC and Sal B-UC groups showed a significantly lower strain rate from the second to the eighth week (*p* < 0.001). In the UC group, once the strain rate had decreased in the mitral lateral segment in the second week, it did not show any further changes. The strain rate decreased in the second week in the middle lateral segment, and further diminished myocardial function was observed in the fourth week (*p* for time = 0.202). In the other five segments, the *p-*value for the time was <0.001, and the strain rate in the UC group decreased compared with the sham group, indicating fluctuating during UC development. The Sal B-UC group showed a similar level of myocardial movement to the UC group, and the strain rate in the Sal B-UC group was significantly lower than in the sham group (*p* < 0.001).

**Table 3:** 2D-speckle tracking echocardiography measurements in rats at 6 and 8 weeks.

**Time**	**6 weeks**			**8 weeks**			***p* of group**	***p* of time**
**Group**	**Sham**	**UC**	**Sal B-UC**	**Sham**	**UC**	**Sal B-UC**		
APS	4.19 ± 0.41	2.97 ± 0.41^*^	3.12 ± 0.51^‡^	4.08 ± 0.48	3.04 ± 0.4^*^	2.68 ± 0.3^‡^	0.000^**^	0.348
MS	11.35 ± 1.12	7.81 ± 1.09^*^	8.3 ± 1.36^‡^	13.51 ± 1.6	8.9 ± 1.17^*^	8.64 ± 0.96^‡^	0.000^**^	0.030^*^
BS	11.97 ± 1.19	7.5 ± 1.05^*^	9.29 ± 1.52^†^^‡^	11.5 ± 1.36	7.08 ± 0.93^*^	7.5 ± 0.84^‡^	0.000^**^	0.098
APL	6.81 ± 0.67	6.55 ± 0.91	6.79 ± 1.11	7.45 ± 0.88	5.12 ± 0.67^*^	7.05 ± 0.79^†^	0.000^**^	0.503
ML	8.65 ± 0.86	6.55 ± 0.91^*^	6.23 ± 1.02^†^	9.23 ± 1.09	6.19 ± 0.81^*^	6.05 ± 0.67^†^	0.000^**^	0.971
BL	10.68 ± 1.06	5.86 ± 0.82^*^	7.48 ± 1.23^†^^‡^	7.72 ± 0.92	7.23 ± 0.95	7.05 ± 0.79	0.000^**^	0.101

*Longitudinal strain rate in the sham, UC, and Sal B-UC groups. Two-way ANOVA was performed to test the difference between groups and time points, and Tukey's post-hoc tests were used for group comparisons. Significance marks were fitted to compare data at each time point*.

* *Indicates a significant difference between the sham and UC groups. The*

** *symbol indicates the significant difference of p < 0.01*.

† *Indicates a significant difference between Sham and UC-Sal B groups*.

‡ *indicates a significant difference between the UC and UC-Sal B groups. The significance level was p < 0.05. APS, strain rate of the apical segment of the septum; MS, strain rate of the middle segment of the septum; BS, strain rate of the basal segment of the septum; APL, strain rate of the apical segment of the lateral free wall; ML, strain rate of the middle segment of the lateral free wall; BL, strain rate of the basal segment of the lateral free wall*.

*In this table, the two-way anova only include the data in 6 and 8 weeks. The full data including 2 and 4 weeks could be seen at Supplementary Table S4*.

### Histopathological Findings

**Figure 2:**
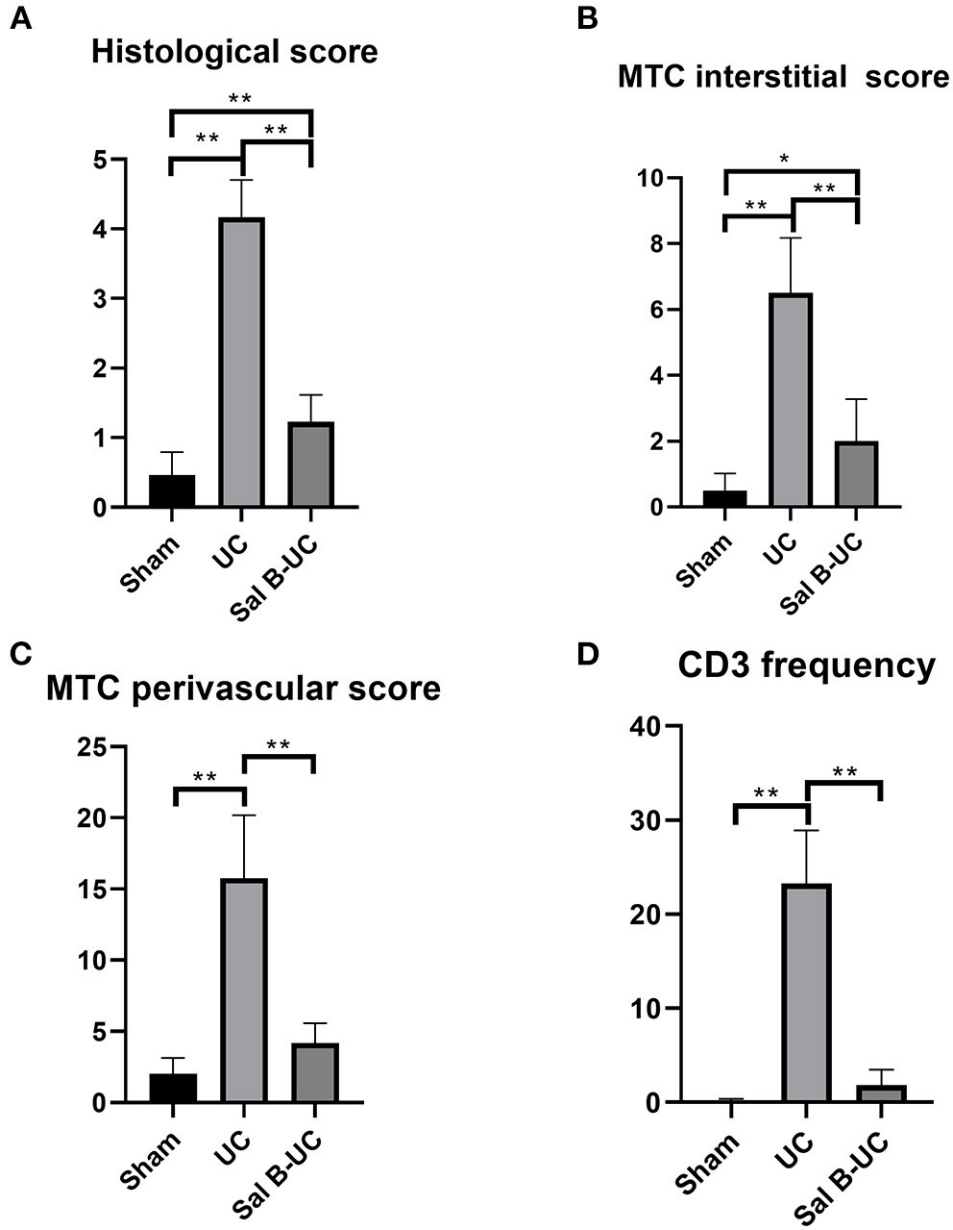
Pathology score evaluation. **(A)** The histological score in the UC group was significantly higher than in the other groups. **(B)** Fibrosis intensity in the UC group was more than in the Sham and Sal B-UC groups as indicated by the blue staining of Masson's trichrome score. **(C)** Fibrosis in the perivascular area in the UC group was also high. **(D)** CD3+ T-lymphocyte frequency in the UC group was the highest among the three groups. The * symbol indicates the significant difference of *p* < 0.05. The ** symbol indicates the significant difference of *p* < 0.01.

**Figure 3:**
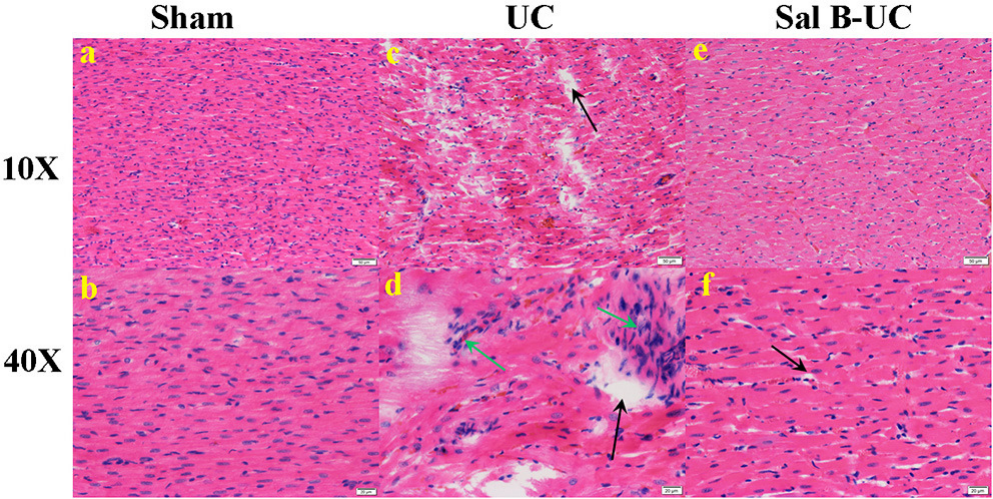
Histological analysis. The histopathological images (10× and 40×) in the sham, UC, and Sal B-UC groups. **(A,B)** Myocytes are arrayed in parallel, with no inflammatory cell infiltration in the sham group. **(C,D)** Distorted cardiomyocytes, mononuclear cell infiltration (green arrow), and interstitial edema with necrosis (black arrow) in the UC group. **(E,F)** Mild edema with little inflammatory cell infiltration in the Sal B-UC group.

**Figure 4:**
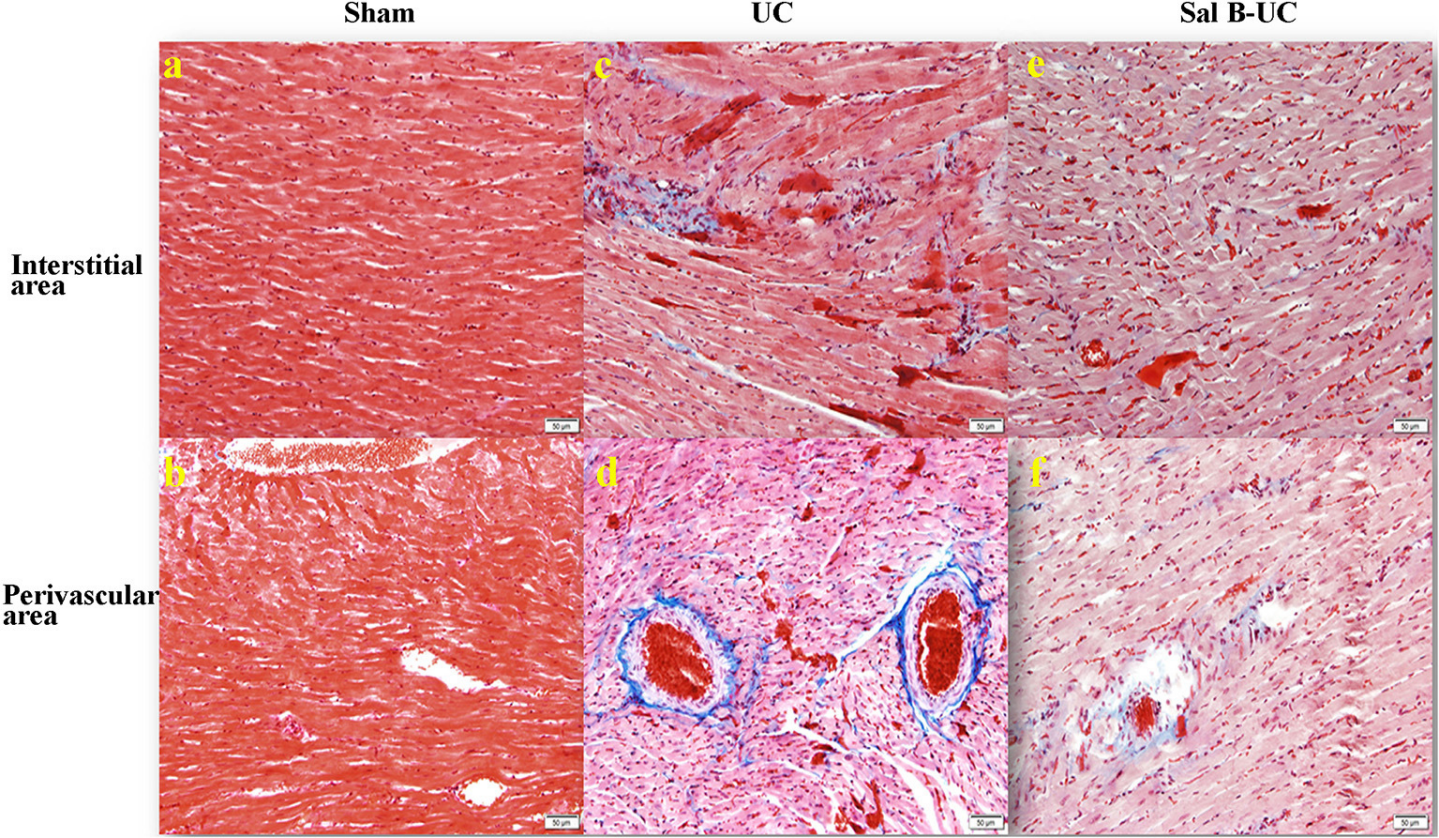
Masson's trichrome staining. The Masson's trichrome images of the interstitial and perivascular area in the sham, UC, and Sal B-UC groups (50 μm). The light blue staining indicates the severity of cardiac fibrosis. In the sham group, fibrosis was not observed at either the interstitial level **(A)** or the perivascular area **(B)**. In the UC group, severe fibrosis was observed at both interstitial levels **(C)** and the perivascular area **(D)**. In the Sal B-UC group, moderate light blue staining was present at the two levels **(E,F)**.

To detect the cellular changes underlying the development of uremic cardiomyopathy in rats, we stained heart tissue with H&E and Masson's trichrome. The histopathological score was significantly higher (*p* < 0.001) in the UC group compared to the sham group (Figure 2A). A significant difference (*p* < 0.001) was observed between the Sal B-UC group and the sham group regarding their histopathological scores (Figure 2B). However, the score in the Sal B-UC group was much lower than the score of the UC group (Figure 2C). And we find CD3+ T-lymphocyte frequency in the UC group was the highest among the three groups (Figure 2D). The inflammatory changes in the UC group were represented in the form of inflammatory cell infiltration (especially mononuclear cell infiltration in Figure 3), severe degeneration and necrosis in cardiomyocytes, and increased size of cardiac myocytes compared to the other groups. Interstitial edema, as well as distorted cardiac muscle fibers, was also clearly observed in the UC group compared with the sham group. Inflammatory cell infiltration and interstitial edema were mild in the Sal B-UC group. Besides, we evaluated fibrosis by using Masson's trichrome to stain the interstitial collagen fibers as well as perivascular collagen in the three groups. A greater intensity of bright blue collagen staining was detected in the UC group compared to the other two groups, as shown in Figure 4, indicating that fibrosis occurred at the interstitial and perivascular levels. In addition, the intensity of fibrosis in the Sal B-UC group was less than in the sham group.

### Immunohistochemical Staining of CD3

**Figure 5:**
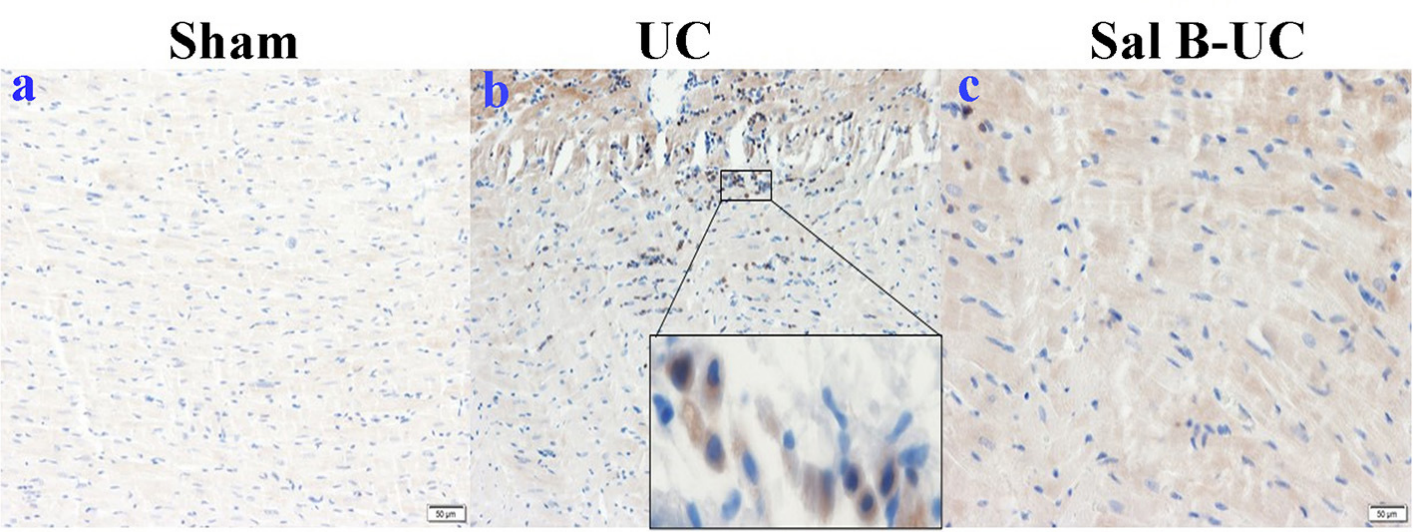
Immunohistochemical (IHC) staining of CD3. The IHC staining of CD3 in the sham, UC, and Sal B-UC groups. CD3-T-lymphocytes were not recognized in the sham group **(A)**. The number of CD3 infiltrations (brown color) was significantly increased in the UC group **(B)**. A limited number of CD3+ was observed in the Sal B-UC group **(C)**.

To characterize the kinetics of inflammatory cell recruitment in uremic cardiomyopathy, an antibody against a marker CD3 (T cell inflammatory marker) was immunohistochemically stained in the three groups. The quantification of CD3+T-lymphocyte infiltration in the UC group was significantly increased (*p* < 0.001) compared to the other groups (Figure 5). Additionally, inflammatory cell recruitment was lower in the Sal B-UC group compared with the sham group. Accumulation of CD3 cells in the heart tissue of UC rats indicated that the molecular mechanism underlying disease progression had occurred.

## Discussion

This study aimed to evaluate cardiac status during the development of UC and assess the treatment effect of Sal B. While chronic kidney disease (CKD), a progressive pathological condition, is a precursor to UC, the pathophysiology of UC is poorly understood. Therefore, we designed this experiment using novel non-invasive techniques (IVPG and 2DSTE) to evaluate cardiac function in UC. These techniques allowed us to track cardiac changes during UC development. Furthermore, we used histopathological analysis to track the pathological changes occurring in UC. The results have given us a deeper understanding of UC pathophysiology. Additionally, we used herbal medicine as a replacement for cardioprotective treatments. The functional and pathological information about Sal B's treatment effect has helped to further our understanding of UC and its management.

Serum concentrations of BUN and creatinine are the gold standard for clinical diagnosis of CKD (25). A 5/6 subtotal nephrectomy is the primary avenue for investigating CKD (26), not only because this model directly decreases functional nephrons, but it is also relevant to clinical patients (21). In the present study, CKD was confirmed in UC rats by elevating BUN and creatinine concentrations from the second week after 5/6 nephrectomy until the endpoint of monitoring. Clinical symptoms like polyuria and hypervolemia support the diagnosis of CKD. Sal B-UC group showed less polyuria and hypervolemia, which indicate Sal B have treatment effect in UC. Sal B alleviate UC by reversing the elevated BUN and creatinine concentrations at 6 and 8 weeks. A Previous study revealed that the impaired kidney function was ameliorated by Sal B *via* a reduction in epithelial-mesenchymal transition-related proteins. Epithelial-mesenchymal transition in renal fibrosis was promoted by Sal B through autophagy activation (27).

We detected multiple types of ventricular geometry in UC. Concentric hypertrophy (elevated RWT and LVM) was observed in UC rats at the two-, 6-, and 8-week time points, but eccentric hypertrophy was also observed at the 4-week time point, evidenced by increased LVM and normalized RWT in the UC rats. This could be explained by the fact that pressure and volume overload dominate at different stages of UC development (28). The consequence of ventricular geometry change in UC makes staging UC based on geometry and intraventricular blood flow challenging, so multiple way analysis becomes necessary for UC diagnosis.

Patients with different types of hypertrophies show differing responses to the same drugs, indicating that different mechanisms are responsible for eccentric and concentric hypertrophy (8). In concentric LV hypertrophy, the sympathetic nervous system is overactivated. The activation of beta-adrenoceptors in the heart results in cardiomyocyte hypertrophy and progressive heart failure (29), which is considered the primary mechanism of concentric hypertrophy (30). The development of eccentric hypertrophy involves mechanical stress and activation of the sympathetic nervous system. The mechanical stress on the LV from pressure overload is stronger than that caused by volume overload (31).

The mechanism by which Sal B is believed to enact its therapeutic effect is similar to that of beta-blockers, which are the first-line therapeutic drugs for concentric hypertrophy. Previously, we have shown that Sal B protects against concentric LV hypertrophy caused by pressure overload and that it prevents further hypertrophy without a pressure-lowering effect (12). In the present study, Sal B showed a potential therapeutic effect against UC. The treatment effect of Sal B was confirmed by preventing the progression of UC because we observed a significantly different histopathology score between the Sal B-UC and sham groups and a dramatic difference in pathology score between the Sal B-UC and UC groups.

Myocardial dysfunction occurred from weeks two through eight. Administration of Sal B did not reverse the decreased longitudinal strain rate at week 6 or 8. The hypertrophy in UC rats was not apparent until week 6, which implies that myocardial dysfunction occurred before the changes in ventricular geometry (32). Furthermore, we found severe edema and inflammation in myocardial tissues and severe interstitial and perivascular fibrosis in the UC group, which confirmed our expectation: myocardial dysfunction was caused by edema, inflammation, and fibrosis in UC. However, edema, inflammation, and fibrosis in the Sal B-UC group were significantly lower than in the UC group (*p* < 0.001), but the strain rate was not significantly different. In other words, the inflammatory reaction was associated with myocardial dysfunction, while the severity of fibrosis was not related to myocardial function in UC. The difference between CD3 positive cells in UC and Sal B-UC indicates that the treatment effect of Sal B decreased the acute inflammatory reaction.

As we mentioned before, UC-associated LV hypertrophy results from complex pressure overload, volume overload, and the uremic state itself. Sal B alleviates UC by improving renal function, decreasing blood pressure, and reducing edema, inflammation, and fibrosis in the myocardial tissue. This pathophysiology mechanism indicates that cardiomyocyte is not the main target of Sal B treatment.

In the current work, resting HR was evaluated under equivalent anesthesia for all rats. Our previous work showed that HR had a weak influence on IVPG, so HR was not intentionally controlled (11). The resting HR in the UC group was higher than in the sham group at every time point following the operation. Elevated resting HR correlates with a higher risk of death (33), which indicates that cardiac function was damaged in the UC group. The impairment was not completely reversed by the Sal B administration.

No differences in IVPG were detected among the groups because IVPG is non-invasive technology concentrating on intracardiac flow, this result indicates that the flow was intact in UC. Basal IVPG correlates with E wave velocity, and mid-to-apical IVPG correlates with myocardial movement (10). Combined with the fact that E wave velocity, E′, and E/E′ also showed no differences between the groups, it is easily concluded that LA pressure was not elevated in the UC group. The usefulness of 2DSTE and IVPG for assessing cardiac function was confirmed in rats before (12, 13, 34). Myocardium dysfunction detected by STE combined with the fact that intracardiac flow was not disturbed in UC to provide us with an interesting conclusion: subtotal nephrectomy induced UC rat model could be used for cardiovascular drug selection. Because only the myocardium was affected in the UC rat model, the effect of medicine designed for myocardium-related diseases could be examined specifically. This information raises a question: did primary and secondary cardiovascular diseases like UC affect the myocardium differently? This will next stage of our research.

The main difference in the mechanism responsible for forming concentric and eccentric hypertrophy is the mechanical stress on the ventricle wall (8, 31). The changes in ventricular morphology indicate that mechanical stress fluctuates during the development of UC. Myocardial dysfunction occurred in the UC group from weeks two through eight, LV mass and RWT fluctuated, eventually leading to a variable but not a significantly different level of active relaxation in the UC group, confirmed by mid-to-apical IVPG.

Previously, scientists documented the structural and functional disorders of UC in mice (35, 36). Hamzaoui et al. pointed out the fact that different outcomes can be observed between two strains of mice emphasizes the importance of carefully comparing conclusions from the scientific literature (35). But the detailed echocardiography parameter and inflammatory markers have never been reported before. To our knowledge, this is the first study to document the detailed structural and functional outcomes of Sal B treatment in UC using echocardiography and histopathology. The data from this paper will be useful in further pharmacological and cardiovascular studies. For the veterinary field, this paper not only provided precious information about the pathophysiology of UC by novel ultrasound technique, but it will also encourage veterinarians to use this novel ultrasound technique in clinical practices in the future.

## Limitations

Detailed molecular investigations were not performed in the current study. CD45 and RNA were not measured, because this research is concentrating on evaluating non-invasive cardiac function in UC.

## Conclusion

Myocardial dysfunction occurs before ventricular morphological changes during the development of UC, and intracardiac flow was not affected in UC. Both eccentric hypertrophy and concentric hypertrophy were observed in UC, while only eccentric hypertrophy was observed in Sal B-treated rats. Sal B alleviates cardiomyopathy and prevents further development in the UC model. This study demonstrated increased ventricular stiffness and fibrosis in UC. Further studies are warranted to clarify the molecular pathways of Sal B for the treatment of UC.

## Data Availability Statement

The datasets presented in this study can be found in online repositories. The names of the repository/repositories and accession number(s) can be found in the article/Supplementary Material.

## Ethics Statement

The animal study was reviewed and approved by Institutional Animal Care and Use Committee of the Tokyo University of Agriculture and Technology.

## Author Contributions

DM: conceptualization, methodology, software, writing—original draft, writing—review and editing, and visualization. AM and AE: methodology, software, and writing—review and editing. TY and KN: resources and data curation. HH: conceptualization and project administration. HE-H: investigation. KT: methodology and software. YZ: data and curation. ZZ and RT: project administration. All authors contributed to the article and approved the submitted version.

## Conflict of Interest

The authors declare that the research was conducted in the absence of any commercial or financial relationships that could be construed as a potential conflict of interest.

## Publisher's Note

All claims expressed in this article are solely those of the authors and do not necessarily represent those of their affiliated organizations, or those of the publisher, the editors and the reviewers. Any product that may be evaluated in this article, or claim that may be made by its manufacturer, is not guaranteed or endorsed by the publisher.
