# Supplementary material for: Changes in Cardiac Function During the Development of Uremic Cardiomyopathy and the Effect of Salvianolic Acid B Administration in a Rat Model
Source: Front Vet Sci. 2022 Jun 16;9:905759. doi: 10.3389/fvets.2022.905759 (PMC9244798; doi:10.3389/fvets.2022.905759)
Supplement: Supplementary file 1 [file Data_Sheet_1.docx]

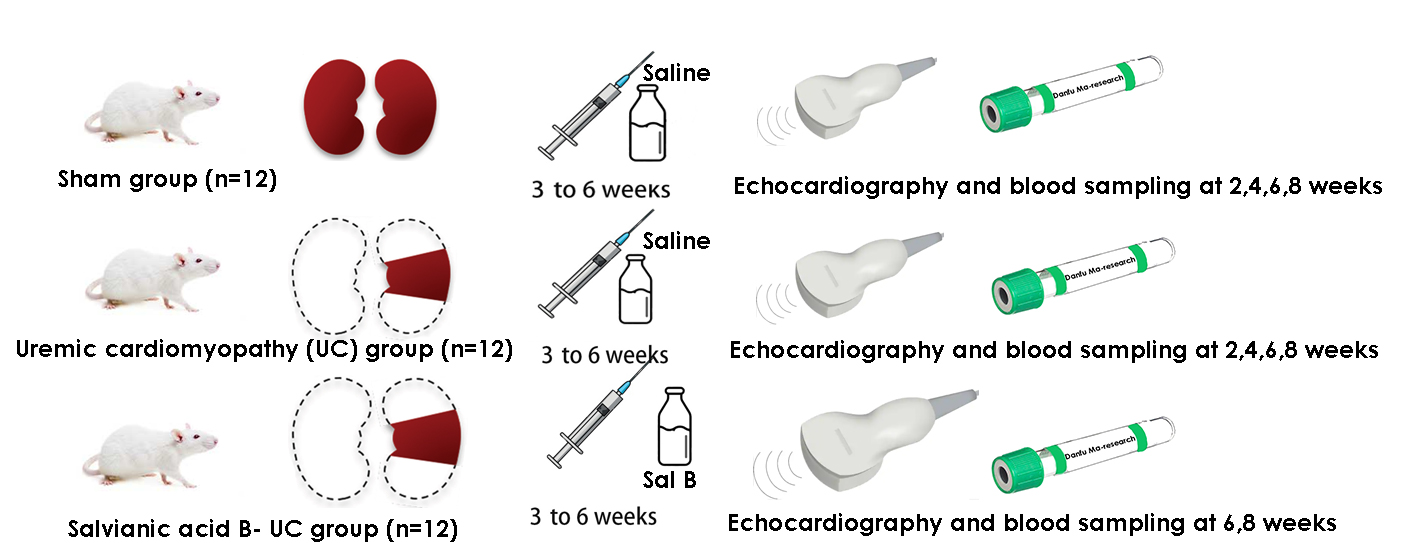


Supplementary figure S1. Schematic illustration of experimental procedures in sham, UC, and Sal B-UC groups.


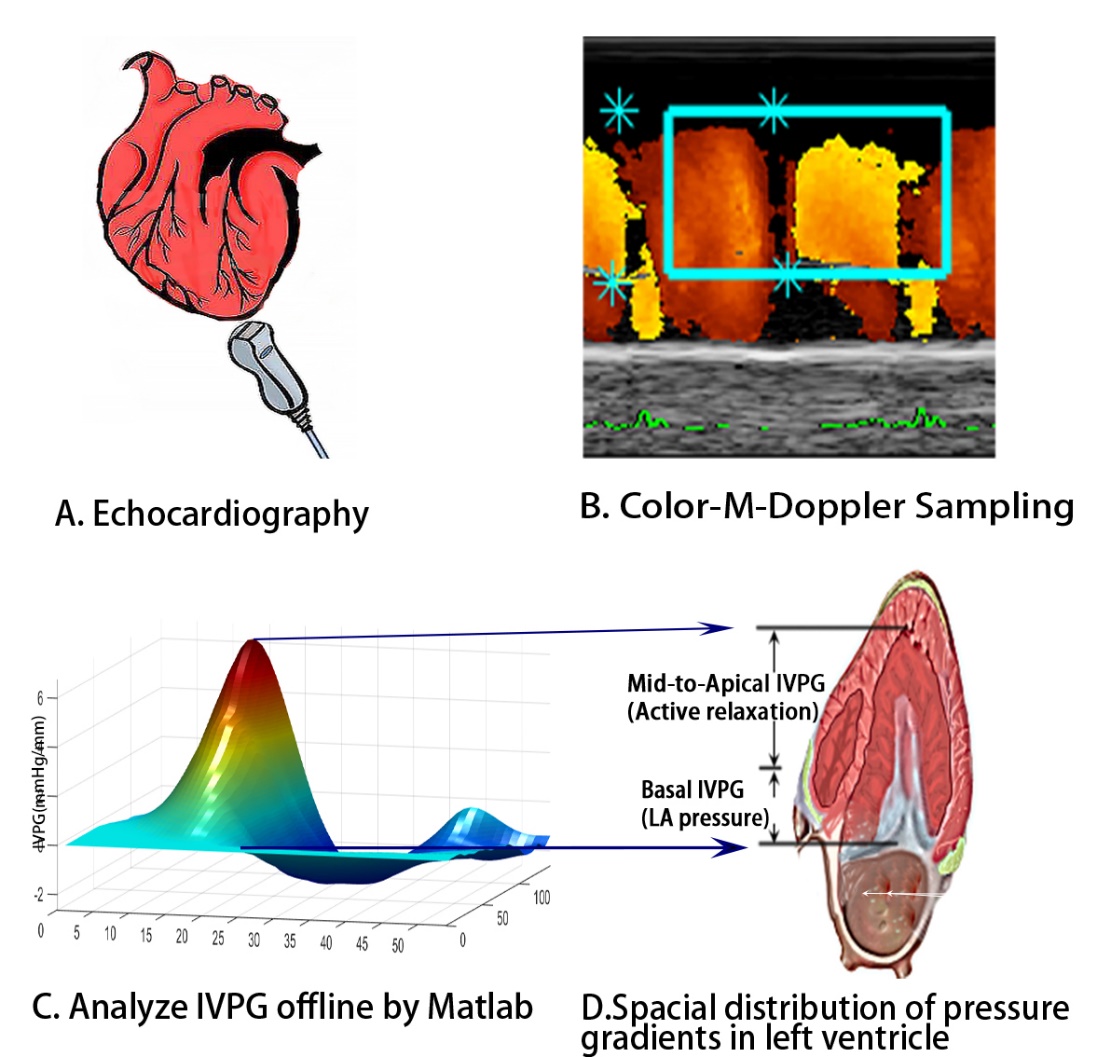


Supplementary figure S2. Measurement of the intraventricular pressure gradients (IVPG) from Euler equation using MATLAB software.


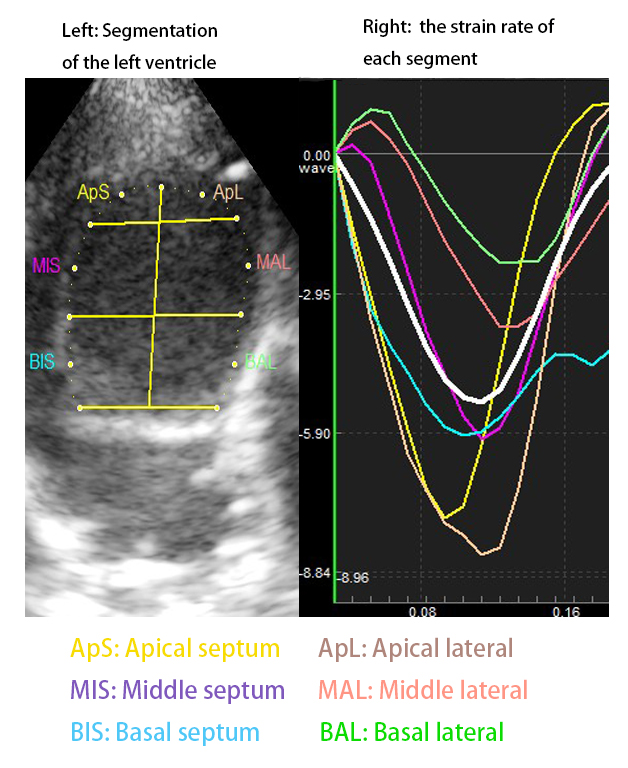


Supplementary figure S3. Speckle tracking echocardiography from left apical view. Left: segmentation of the left ventricle. APS, the apical segment of the septum; MS, the middle segment of the septum; BS, the basal segment of the segment; APL, the apical segment of the lateral free wall; ML, the middle segment of the lateral free wall; BL, the basal segment of the lateral free wall. Right: the strain rate of each segment.

**Table S1. Cardiac morphology, hemodynamics, and speckle tracking echocardiography in sham and UC groups at two and four weeks**

| Time | 2 weeks | | 4 weeks | | *p* of time | *p* of group |
| --- | --- | --- | --- | --- | --- | --- |
| Group | Sham | UC | Sham | UC |  |  |
| **A. Cardiac morphology** | | | | | | |
| IVSs | 1.88±0.28^*^ | 2.57±0.43^*^ | 1.79±0.32^*^ | 2.21±0.48^*^ | 0.042^*^ | 0.000^**^ |
| LVIDs | 4.19±0.42^*^ | 3.74±0.4^*^ | 4.38±0.56 | 4.25±0.86 | 0.028^*^ | 0.029^*^ |
| LVPWs | 2.27±0.27 | 2.51±0.4 | 2.21±0.34 | 2.24±0.16 | 0.001^**^ | 0.009^**^ |
| IVSs | 1.88±0.28^*^ | 2.57±0.43^*^ | 1.79±0.32^*^ | 2.21±0.48^*^ | 0.042^*^ | 0.000^**^ |
| IVSd | 1.12±0.1 | 1.5±0.31^*^ | 1.13±0.12 | 1.36±0.22^*^ | 0.002^**^ | 0.000^**^ |
| LVIDd | 7.36±0.72 | 6.58±1.47^*^ | 7.71±0.68 | 7.67±0.55 | 0.022^*^ | 0.024^*^ |
| LVPWd | 1.43±0.25 | 1.72±0.35 | 1.4±0.21 | 1.43±0.26 | 0.089 | 0.019^*^ |
| FS | 42.54±4.46 | 46.63±4.29 | 43.22±4.55 | 44.97±8.2 | 0.461 | 0.292 |
| LVM | 0.63±0.08 | 0.68±0.13 | 0.64±0.07 | 0.73±0.15^*^ | 0.004^**^ | 0.000^**^ |
| HW | 0.85±0.09 | 0.87±0.10 | 0.86±0.08 | 0.98±0.16 | 0.003^**^ | 0.000^**^ |
| HW/BW | 3.62±0.19 | 3.91±0.21 | 3.81±0.23 | 4.61±0.25 | 0.003^**^ | 0.000^**^ |
| RWT | 0.35±0.07 | 0.46±0.09^*^ | 0.33±0.05 | 0.38±0.07 | 0.012^*^ | 0.000^**^ |
| **B. Hemodynamic data** | | | | | | |
| SAP | 104.14±10.68 | 115.86±5.59^*^ | 103.21±5.19 | 124.63±12.6^*^ | 0.034^*^ | 0.000^**^ |
| HR | 330.49±32.05 | 358.33±29.3 | 310.71±35.46 | 346.34±38.94^*^ | 0.425 | 0.009 |
| DAP | 78.6±18.95 | 80.29±4.49 | 74.26±6.38 | 98.48±10.75^*^ | 0.000^**^ | 0.000^**^ |
| MAP | 87.11±15.24 | 92.15±2.43 | 83.91±4.91^*^ | 107.2±9.64^*^ | 0.000^**^ | 0.000^**^ |
| E | 99.58±13.48 | 98.5±13.39 | 91.23±10.82 | 110.57±8.85^*^ | 0.447 | 0.003 |
| E’ | 5.69±0.65 | 6.04±1.23 | 5.71±0.66 | 5.57±0.39 | 0.804 | 0.510 |
| **C. 2D-speckle tracking echocardiography** | | | | | | |
| APS | 3.83±0.41 | 2.39±0.3^*^ | 3.72±0.37 | 2.58±0.47^*^ | 0.346 | 0.000 |
| MS | 14.64±1.58 | 6.89±0.8^*^ | 13.58±1.36 | 8.8±1.38^*^ | 0.037 | 0.000 |
| BS | 10.28±1.11 | 7.5±0.9^*^ | 18.84±1.88 | 7.07±1.14^*^ | 0.000 | 0.000 |
| APL | 7.96±0.86^*^ | 6.21±0.94^*^ | 6.31±0.63 | 6.1±0.87 | 0.042 | 0.000 |
| ML | 10.27±1.11 | 5.92±0.66^*^ | 9.65±0.96 | 6.32±0.64^*^ | 0.081 | 0.000 |
| BL | 9.02±0.97 | 7.78±1.0^*^ | 8.02±0.8 | 5.01±0.68^*^ | 0.000 | 0.000 |

Two-way ANOVA was performed to test the difference between groups and time points, and Tukey’s post hoc tests were used for group comparisons. Significance marks were fitted to compare data at each time point. * indicates a significant difference between the sham and UC groups at each time point. The significance level was p < 0.05. IVSd, interventricular septum diastolic diameter; LVIDd, left ventricular internal diastolic diameter; LVPWd, left ventricular posterior wall diastolic diameter; FS, fraction shorting; LVM, left ventricle mass; RWT, relative wall thickness; IVSs, interventricular septum systolic diameter; LVIDs, left ventricular internal systolic diameter; LVPWs, left ventricular posterior wall systolic diameter; FS, fraction shorting; LVM, left ventricle mass; HW, heart weight(g); HW/BW, heart weight(mg)/body weight(g); WT, relative wall thickness; HR, heart rate; SAP, systolic arterial pressure; DAP, diastolic arterial pressure; MAP, mean arterial pressure; E’, Peak velocity of early diastolic mitral annular motion as determined by pulsed-wave Doppler; APS, strain rate of the apical segment of the septum; MS, strain rate of the middle segment of the septum; BS, strain rate of the basal segment of the septum; APL, strain rate of the apical segment of the lateral free wall; ML, strain rate of the middle segment of the lateral free wall; BL, strain rate of the basal segment of the lateral free wall.

**Table S2. Percentage change of BUN and creatinine**

|  | BUN | | | | Creatinine | | | |
| --- | --- | --- | --- | --- | --- | --- | --- | --- |
|  | Sham | UC | Sal B-UC | *p* of group | Sham | UC | Sal B-UC | *p* of group |
| 2 to 4 weeks | 2.56±0.71 | 4.17±0.92^*^ | 10.36±3.29^*†^ | 0.000^**^ | -5.5±1.87 | -21.49±3.25^*^ | -17.38±4.42^*†^ | 0.000^**^ |
| 4 to 6 weeks | -5±1.81 | 2.4±0.36^*^ | -13.88±4.64^*†^ | 0.000^**^ | 2.56±0.91 | -5.77±1.25^*^ | 29.1±6.79^*†^ | 0.000^**^ |
| 6 to 8 weeks | 6.58±2.02 | 2.86±1.51^*^ | -1.82±0.51^*†^ | 0.000^**^ | 7.08±2.84 | 2.14±0.68^*^ | 1.57±0.4^*^ | 0.000^**^ |

Two-way ANOVA was performed to test the difference between groups and time points, and Tukey’s post hoc tests were used for group comparisons. Significance marks were fitted to compare data at each time point. * indicates a significant difference between the sham and UC, sham and Sal B-UC groups. † indicates a significant difference between UC and UC-Sal B groups. The significance level was p < 0.05. The percentage change was calculated by the formula: (initial value-new value)/initial value, so the percentage change is less than zero indicating the new value is higher than the initial value.
