# Supplementary material for: Changes in Cardiac Function During the Development of Uremic Cardiomyopathy and the Effect of Salvianolic Acid B Administration in a Rat Model
Source: Front Vet Sci. 2022 Jun 16;9:905759. doi: 10.3389/fvets.2022.905759 (PMC9244798; doi:10.3389/fvets.2022.905759)
Supplement: Supplementary file 2 [file Data_Sheet_2.docx]

| time | 2 weeks | | 4 weeks | | 6 weeks | | | 8 weeks | | | *p* of time | *p* of group |
| --- | --- | --- | --- | --- | --- | --- | --- | --- | --- | --- | --- | --- |
| Group | sham | UC | sham | UC | sham | UC | Sal B-UC | sham | UC | Sal B-UC |  |  |
| SAP | 104.14±10.68^#^ | 115.86±5.59^*^ | 103.21±5.19^#^ | 124.63±12.6^*^ | 93.41±15^#^ | 133.58±13.56^*^ | 121.24±9.45^#^ | 100.53±15.25^#^ | 144.12±11.98^*^ | 126.75±11.21^*#^ | 0.002^**^ | 0.000^**^ |
| DAP | 78.6±18.95 | 80.29±4.49 | 74.26±6.38^#^ | 98.48±10.75^*^ | 73.75±6.55^#^ | 104.13±9.8^*^ | 88.59±9.82^#^ | 73.2±5.63^#^ | 123.93±19.57^*^ | 106.18±14.43^*#^ | 0.000^**^ | 0.000^**^ |
| MAP | 87.11±15.24 | 92.15±2.43 | 83.91±4.91^#^ | 107.2±9.64^*^ | 80.3±6.17^#^ | 113.94±8.08^*^ | 110.36±7.15 | 82.31±8.05^#^ | 130.66±13.52^*^ | 119.89±8.4^*#^ | 0.000^**^ | 0.000^**^ |
| IVSd | 1.12±0.1^#^ | 1.5±0.31^*^ | 1.13±0.12^#^ | 1.36±0.22^*^ | 1.21±0.17^#^ | 1.57±0.35^*^ | 1.39±0.26 | 1.3±0.16^#^ | 1.67±0.3^*^ | 1.57±0.15^*^ | 0.001^**^ | 0.000^**^ |
| LVIDd | 7.36±0.72^#^ | 6.58±1.47^*^ | 7.71±0.68 | 7.67±0.55 | 7.53±0.62 | 7.32±0.27 | 7.93±0.5 | 7.25±0.58^#^ | 7.32±0.54 | 7.87±0.69 | 0.017^*^ | 0.000^**^ |
| LVPWd | 1.43±0.25 | 1.72±0.35 | 1.4±0.21 | 1.43±0.26 | 1.32±0.27^#^ | 1.78±0.54^*^ | 1.43±0.36^#^ | 1.64±0.33 | 1.95±0.53 | 1.49±0.46 | 0.016^*^ | 0.000^**^ |
| IVSs | 1.88±0.28^#^ | 2.57±0.43^*^ | 1.79±0.32^#^ | 2.21±0.48^*^ | 2.06±0.39 | 2.29±0.55 | 2.16±0.46 | 1.96±0.4^#^ | 2.5±0.52^*^ | 2.63±0.19^*^ | 0.042^*^ | 0.000^**^ |
| LVIDs | 4.19±0.42^#^ | 3.74±0.4^*^ | 4.38±0.56 | 4.25±0.86 | 4.34±0.5 | 4.25±0.38 | 4.48±0.36 | 4.36±0.46 | 3.93±0.51 | 4.13±0.44 | 0.028^*^ | 0.029^*^ |
| LVPWs | 2.27±0.27 | 2.51±0.4 | 2.21±0.34 | 2.24±0.16 | 2.32±0.32 | 2.55±0.72 | 2.39±0.18 | 2.38±0.43^#^ | 2.88±0.68^*^ | 2.95±0.44^*^ | 0.001^**^ | 0.009^**^ |
| FS | 42.54±4.46 | 46.63±4.29 | 43.22±4.55 | 44.97±8.2 | 42.59±4.63 | 41.78±4.99 | 43.58±4.19 | 39.59±7.6^#^ | 46.45±5.41^*^ | 47.67±3.28^*^ | 0.185 | 0.003^**^ |
| LVM | 0.6±0.08 | 0.68±0.13 | 0.64±0.07 | 0.73±0.15 | 0.62±0.11^#^ | 0.91±0.34^*^ | 0.79±0.23 | 0.71±0.11 | 0.99±0.38 | 0.86±0.24 | 0.008^**^ | 0.000^**^ |
| RWT | 0.35±0.07^#^ | 0.46±0.09^*^ | 0.33±0.05 | 0.38±0.07 | 0.34±0.07^#^ | 0.46±0.13^*^ | 0.36±0.07^#^ | 0.41±0.07 | 0.49±0.09 | 0.39±0.06^#^ | 0.000^**^ | 0.000^**^ |

Table S3. The cardiac morphology and data of Sham operation, UC, and SalB treated rats

Echocardiographic measurements in sham, UC, and Sal B-UC group rats. Two-way ANOVA was performed to test the difference between groups and time points, Turkey test was used for post hoc comparison. SAP, systolic arterial pressure; DAP, diastolic arterial pressure; MAP, mean arterial pressure; IVSd, interventricular septum diastolic diameter; LVIDd, left ventricular internal diastolic diameter; LVPWd, left ventricular posterior wall diastolic diameter; IVSs, interventricular septum systolic diameter; LVIDs, left ventricular internal systolic diameter; LVPWs, left ventricular posterior wall systolic diameter; FS, fraction shorting; LVM, left ventricle mass; RWT, relative wall thickness.

* and # were used for consistent subset classification, * indicates a significant difference with sham operation rats, and # indicates a significant difference with the UC group. *p* < 0.05 was considered as the significance level.

| time | 2 weeks | | 4 weeks | | 6 weeks | | | 8 weeks | | | *p* of time | *p* of group |
| --- | --- | --- | --- | --- | --- | --- | --- | --- | --- | --- | --- | --- |
| Group | sham | UC | sham | UC | sham | UC | Sal B-UC | sham | UC | Sal B-UC |  |  |
| E | 99.58±13.48 | 98.5±13.39 | 91.23±10.82^#^ | 110.57±8.85^*^ | 105.58±17.51 | 106.49±10.79 | 103.26±14.52 | 97.71±16.91 | 106.47±16.2 | 99.83±19.44 | 0.351 | 0.054 |
| HR | 330.49±32.05 | 358.33±29.3 | 310.71±35.46 | 346.34±38.94 | 298.74±47.14^#^ | 378±56.29^*^ | 353.03±42.92^*^ | 310.23±50.05^#^ | 375.72±45.1^*^ | 364.51±45.82^*^ | 0.510 | 0.000^**^ |
| E’ | 5.69±0.65 | 6.04±1.23 | 5.71±0.66 | 5.57±0.39 | 5.18±1.06^#^ | 6.32±1.42^*^ | 6.06±1.09^*^ | 5.48±0.6 | 6.16±0.77 | 5.62±0.53 | 0.799 | 0.024 |
| APS | 3.83±0.41^#^ | 2.39±0.3^*^ | 3.72±0.37^#^ | 2.58±0.47^*^ | 4.19±0.41^#^ | 2.97±0.41^*^ | 3.12±0.51^*^ | 4.08±0.48^#^ | 3.04±0.4^*^ | 2.68±0.3^*^ | 0.000^**^ | 0.000^**^ |
| MS | 14.64±1.58^#^ | 6.89±0.8^*^ | 13.58±1.36^#^ | 8.8±1.38^*^ | 11.35±1.12^#^ | 7.81±1.09^*^ | 8.3±1.36^*^ | 13.51±1.6^#^ | 8.9±1.17^*^ | 8.64±0.96^*^ | 0.000^**^ | 0.000^**^ |
| BS | 10.28±1.11^#^ | 7.5±0.9^*^ | 18.84±1.88^#^ | 7.07±1.14^*^ | 11.97±1.19^#^ | 7.5±1.05^*^ | 9.29±1.52^*#^ | 11.5±1.36^#^ | 7.08±0.93^*^ | 7.5±0.84^*^ | 0.000^**^ | 0.000^**^ |
| APL | 7.96±0.86^#^ | 6.21±0.94^*^ | 6.31±0.63 | 6.1±0.87 | 6.81±0.67 | 6.55±0.91 | 6.79±1.11 | 7.45±0.88^#^ | 5.12±0.67^*^ | 7.05±0.79^#^ | 0.003^**^ | 0.000^**^ |
| ML | 10.27±1.11^#^ | 5.92±0.66^*^ | 9.65±0.96^#^ | 6.32±0.64^*^ | 8.65±0.86^#^ | 6.55±0.91^*^ | 6.23±1.02^*^ | 9.23±1.09^#^ | 6.19±0.81^*^ | 6.05±0.67^*^ | 0.202 | 0.000^**^ |
| BL | 9.02±0.97^#^ | 7.78±1^*^ | 8.02±0.8^#^ | 5.01±0.68^*^ | 10.68±1.06^#^ | 5.86±0.82^*^ | 7.48±1.23^*#^ | 7.72±0.92 | 7.23±0.95 | 7.05±0.79 | 0.000^**^ | 0.000^**^ |

Table S4. Echocardiographic measurements and longitudinal strain rate in uraemic cardiomyopathy, sham operation, and Sal B-treated rats

Echocardiographic measurements and longitudinal strain rate in sham, UC, and Sal B-UC group rats. Two-way ANOVA was performed to test the difference between groups and time points, Turkey test was used for post hoc comparison. E, the velocity of early mitral inflow; HR, heart rate; E’, Peak velocity of early diastolic mitral annular motion as determined by pulsed-wave Doppler; E/E’. in sham, UC, and Sal B-UC group rats. APS, strain rate of the apical segment of the septum; MS, strain rate of the middle segment of the septum; BS, strain rate of the basal segment of the septum; APL, strain rate of the apical segment of the lateral free wall; ML, strain rate of the middle segment of the lateral free wall; BL, strain rate of the basal segment of the lateral free wall.

* and # were used for consistent subset classification, * indicates a significant difference with sham operation rats, and # indicates a significant difference with the UC group. *p* < 0.05 was considered as the significance level.
